# Sutureless Aortic Valves in combined procedures: a useful tool in the armamentarium of cardiac surgeons

**DOI:** 10.1186/1749-8090-10-S1-A315

**Published:** 2015-12-16

**Authors:** Steffen Pfeiffer, Ferdinand Vogt, Joachim Sirch, Theodor Fischlein, Giuseppe Santarpino

**Affiliations:** 1 Department of Cardiac Surgery - Paracelsus Medical University Nuremberg - Germany

## Background/Introduction

Following the encouraging preliminary results, sutureless aortic valve implantation is performed in a growing number of patients as it makes minimally invasive surgery easier.

## Aims/Objectives

On the other hand, less data are available on the performance of sutureless aortic valves in combined or complex procedures.

## Method

Between May 2010 and May 2015, 319 patients (age 77.4 ± 5 years, female 169 (53%) underwent aortic valve replacement with a sutureless bioprosthesis in our institution. Of them, 25 were operated upon as REDO (10 with a degenerated aortic bioprosthesis and/or 15 with previous CABG) or as combined procedures (114 Patients, Table 1). In-hospital and follow up clinical and echocardiographic data were collected for all patients and here reported for the combined procedures.

## Results

Mean logistic EuroScore was 14.7 ± 12%. The patients received a size S (n = 4), M (n = 40), L (n = 53) or XL (n = 17) prosthesis. Mean aortic cross-clamp time and cardiopulmonary bypass time were 55.3 ± 21 and 88.8 ± 29 minutes, respectively. In-hospital mortality was 6.1%. We recorded 15 pacemaker implantations (13.1%). At follow-up (27 ± 24 months), we observed 2 pts. with endocarditis needing reoperation, 1 of these died postoperatively. Mean transprosthetic gradients were 13.4 ± 5, 13.8 ± 4.5, 13.7 ± 6.4 at 6 months, 1 year, and 2 years, respectively.

## Discussion/Conclusion

The sutureless aortic valve represents a useful tool in the armamentarium of cardiac surgeons for combined and complex surgery. As with growing experience, the indications and the limitations may become the same as for a conventional biological prosthesis but its use can make the operations faster, especially in complex and long procedures.

